# The History of Syphilis

**Published:** 1891-12

**Authors:** 


### THE HISTORY OF SYPHILIS.

Seven towns disputed about the birthplace of Homer, each claiming the honor. Three nations have disputed about the birthplace of Syphilis, each modestly declining the doubtful compliment of having inoculated the world with this *grosse verole.*

It was unknown in Europe prior to 1494. Toward the close of this year the armies of Charles VIII., King of France, invaded Italy and occupied Naples for a few months. Whether by coincidence or not, no one can say, but it was during this time that the new disease made its unwelcome appearance. The Neapolitans called it the "French Disease," and the invaders called the new comer the "Disease of Naples."

Again, another historical event is adduced to which some trace the origin of the disease. Columbus and his men, after their memorable first visit to America, which is to be duly celebrated in Chicago shortly, returned to Spain in 1493. In addition to the important historical and geographical achievement of discovering America, it is said that they discovered (*nolens volens*) something else over here which has never yet been of any manifest benefit to mankind. Their new find was syphilis, and along with other relics and treasures, they bore it back to the Old World, and industriously began the work of its rapid dissemination. In twelve months it had become an epidemic.

In the August issue of the *American Journal of Medical Science*, Dr. Hyde, of Chicago, contributes a paper on Pre Columbian Syphilis in America, in which the history of the disease is reviewed, and the theory of its American origin very ably discussed. A valuable contribution to this subject has been made by Prof. Joseph Jones (*New Orleans Medical and Surgical*

*Journal*, June, 1878), who examined the skeletons found in Indian mounds in Mississippi, Tennessee and Kentucky, and concluded that he had found the "most ancient syphilitic bones in the world." From etymological studies Dr. Bruhl, of Cincinnati, believes that the disease existed among the Indians before the advent of Spaniards.

Dr. Hyde has carefully examined some bones exhumed from mounds in Arkansas, California, Colorado, etc., and he notes that there is a "conspicuous absence of certain features which are to be expected in bone syphilis. There were no nodules, nor the circumscribed swellings at the distal extremities of syphilitic bones, nor circumscribed or diffuse gummatous involvement, nor bone cicatrices, nor localized sclerosis. Knowing nothing of therapeutics the aborigines would certainly have transmitted to their offspring unmistakable signs of the disease. Yet Dr. Hyde looked in vain for the lesions of inherited syphilis. He, furthermore, submitted two tibiæ to Dr. T. M. Prudden, of the laboratory of the College of Physicians and Surgeons, New York, for microscopical examination. Prof. Prudden concludes: "That the individual was not the victim of any phase of hereditary syphilis, which induced developmental malformations of these bones, is evident from the well formed articular extremities. On the whole, while I am disposed to think that there is nothing in the morphological condition of these bones which would forbid the assumption that the lesions might have been induced by the atypical form of syphilitic inflammation, they present, nevertheless, no morphological evidence to justify such a belief."

From the examination of bones, hitherto made, from the mounds of the Cave-dwellers of North America, it is "not proven beyond question that said bones are either syphilitic or prehistoric. And it is not difficult to conclude that positive proof of the prevalence of syphilis among the prehistoric races of America, based upon osseous changes, is scarcely yet at hand.

By all the preceding we are reminded of the remark of the great French wit: "Syphilis is like the fine-arts; we do not know the inventor of it."*

**La verole est comme les beaux-arts; on ignore quel en a ete l'inventeur.— Voltaire.*

